# Harnessing host innate immunity may combat acquired resistance to BRAFi

**DOI:** 10.1186/1479-5876-12-S1-O10

**Published:** 2014-05-06

**Authors:** Thomas Tan, Pradeepa Pangigadde, Francesco Sabbatino, Elvira Favoino, Laura Orgiano, Soldano Ferrone, Ennio Carbone, Francesco Colucci

**Affiliations:** 1 School of Clinical Medicine, University of Cambridge, United Kingdom; 2 Karolinska Institute, Stockholm, Sweden and Università di Catanzaro Magna Graecia, Italy; 3 Massachusetts General Hospital, Boston, United States

## Background

Natural Killer (NK) cells are innate immunity lymphocytes that spontaneously recognise and kill melanoma cells [1]. Dacarbazine synergises with host immunity by inducing stress signals (NKG2D-L) that alert NK cells [2]. This provides an example of how cancer therapies and host innate immunity may be combined to improve management of melanoma patients. BRAF inhibitors (BRAFi) are radically changing clinical practice in melanoma treatment because they offer unprecedented survival gains. However, melanoma patients nearly always relapse with drug-resistant disease after 6-12 months into the treatment. While evidence is emerging that BRAFi may modulate host immunity, the effect of BRAFi on the cross talk between NK cells and melanoma cells is unknown.

## Material and methods

BRAF-mutant melanoma cell lines 1520, SK-MEL-37 and Colo38 were cultured for 4 weeks in the presence of 5μM vemurafenib. BRAFi-resistant variants were sequenced to confirm the presence of the BRAF^V600E^ mutation and exclude outgrowth of BRAF^WT^ clones. NK cells were isolated from the blood of healthy donors and activated with IL-2. Melanoma cell killing by NK cells was quantified before and after BRAFi treatment by FACS (CD107 degranulation assay) and/or by chromium release assays. MAPK signalling in melanoma cells was quantified by FACS (phospho-ERK). Expression of HLA class I, TRAIL, PD-1L as well as ligands for NKG2D, NCRs and DNAM-1 on melanoma cells was assessed by FACS. Receptor expression was assessed by FACS on blood NK cells from healthy donors or by confocal microscopy in lymph node biopsies of metastatic melanoma patients.

## Results

Melanoma cells that acquired BRAFi resistance through increased MAPK signalling developed also resistance towards NK cell killing. Expression of HLA class I, PD-1L and TRAIL was often elevated in drug-resistant melanoma cells compared to parental melanoma cells, whereas the expression of other NK receptor ligands did not significantly change. PD-1 was found on a subset of NK cells from some melanoma patients and on IL-2 activated NK cells from some healthy donors.

## Conclusion

These data suggest that the PD-1/PD-L1 pathway is an attractive target to restore NK cell activity towards melanoma cells in some patients, thus harnessing host innate immunity to combat acquired BRAFi-resistance.
